# How Do Different Ingredients and Additives Affect the Production Steps and the Bioactive Potential of Mead?

**DOI:** 10.17113/ftb.61.02.23.7622

**Published:** 2023-06

**Authors:** Larissa Simão, Bruna Rafaela da Silva Monteiro Wanderley, Michelly Pontes Tavares Vieira, Isabel Cristina da Silva Haas, Renata Dias de Mello Castanho Amboni, Carlise Beddin Fritzen-Freire

**Affiliations:** Department of Food Science and Technology, Federal University of Santa Catarina, Rod. Admar Gonzaga 1346 Florianópolis, Santa Catarina, Brazil

**Keywords:** honey, mead production, phenolic compounds, alcoholic fermentation, fruit beverage, antioxidant

## Abstract

Mead is a fermented alcoholic beverage that is made from honey diluted in water and commonly with the addition of other ingredients. The chemical characteristics of mead are closely related to the ingredients and additives that are used in its preparation, especially the type of honey, yeast strain and prefermentation nutrients, as well as herbs, spices and/or fruits. These additives can affect not only the fermentation process, in particular the yeast activity, the formation of metabolites and fermentation time, but also the bioactive potential of the mead, which mainly depends on phenolic compounds. Scientific studies have shown that the mead with added different plant species contains considerable amounts of different classes of polyphenols, which have important biological activities. Within this context, this review study seeks to investigate how different ingredients and additives can affect each of the stages of the preparation of mead, as well as its bioactive potential, in order to understand the effects on its chemical composition, and thus add greater commercial value to this beverage.

## INTRODUCTION

Mead is an alcoholic beverage obtained from the fermentation of honey diluted in water (*1*, *2*). Other ingredients such as herbs, spices and/or fruits, are commonly added to modify the chemical and sensory characteristics of this beverage (*3*). The chemical composition of mead can be altered by different factors, such as the origin of the honey, the yeast strains used, and the additives and other ingredients that are incorporated into the wort, as well as the product preparation steps (*4*, *5*).

Although mead has been produced in various regions of the world since ancient times, mainly in Nordic countries and Eastern Europe, in recent years this beverage has shown an important growth in the global alcoholic beverage market (*6*). However, mead is still mostly produced empirically and as a handcrafted beverage, with relatively few scientific reports in comparison with other alcoholic beverages (*7*). Moreover, the production of mead shows some problems related to slow fermentation, mainly due to the variability in the composition of honey and its low buffering capacity, as well as to the limited amount of nutrients in the wort, which are essential for yeast development (*6*, *8*).

The ingredients used for the preparation of the beverage have a significant impact on the fermentation process and can modify the bioactive potential of the mead (*9*, *10*). Studies of the characteristics of meads with added herbs and fruits have been published recently (*3*, *9*, *11*). Besides, nutritional additives and supplements, mainly those that are nitrogen-based, minerals, vitamins and acids have been incorporated into the wort in order to stimulate yeast growth and consequently improve the fermentation process (*7*, *12*, *13*).

Given the above, this review seeks to investigate how different ingredients and additives can affect the stages of the preparation of mead, as well as its bioactive potential, in order to understand the effects on its chemical composition, and thus add greater commercial value to this ancient yet little researched beverage.

## TYPES OF MEAD AND MAIN INGREDIENTS

Traditionally, mead is made from three basic ingredients: water, honey and yeast. However, in order to diversify the beverage and improve its bioactive and sensory properties, different types of honey and yeast can be used, besides other additional ingredients. According to Starowicz and Granvogl (*14*), different meads can be obtained by using different ratios of honey and water, as well as by the addition of fruits, herbs and/or spices or other additives. Thus, there are some denominations regarding the types of mead according to the used ingredients, such as: melomel (addition of fruits or fruit juice) (*15*), cyser (addition of apple juice or cider) (*7*), pyment (addition of grape juice or wine) (*16*), and metheglin (addition of spices and/or herbs) (*7*).

Among the basic ingredients of mead, water is predominant, and thus it is important to check its quality for the preparation of the beverage. In order for water to be used in the manufacture of alcoholic beverages, it must meet certain requirements, such as not having turbidity or high levels of chlorine, having a controlled pH, and meeting the desired microbiological standards (*15*, *17*).

Honey is a natural food which is produced mainly by the honey bee from the nectar of flowers, secretions from living parts of plants, or excretions from sucking insects (*18*, *19*). It is a saturated solution of sugars, mainly fructose (38 %) and glucose (31 %), as well as minor constituents, such as minerals, proteins, amino acids, enzymes, organic acids, aromatic substances, vitamins, phenolic acids, and flavonoids, among others (*20*, *21*). The chemical composition and the sensory characteristics of honey are affected by its botanical and geographic origin, the climatic conditions, maturation stage, bee species, as well as the processing and storage conditions (*20*, *22*). Honey has been acknowledged as a functional food mainly because of its antioxidant and antimicrobial properties (*23*-*25*), and its colour is directly related to its phenolic content (*26*). Thus, honey can be used as a basic raw material for obtaining different foods and beverages, including alcoholic beverages such as mead, and it directly affects the chemical and sensory characteristics of the product (*14*). Floral honey types are used the most for the preparation of mead (*1*, *9*, *10*). However, honeydew honey has aroused the interest of researchers because of its distinct chemical composition (*3*).

The choice of fermentative microorganisms is also of great importance in the production of mead as they play a key role in the efficient conversion of sugar into ethanol (*5*). Yeasts are facultative unicellular and anaerobic microorganisms and are responsible for the fermentation process of mead, especially those of the genus *Saccharomyces*. The species that are best known for their performance in alcoholic beverage fermentations are *S. cerevisiae* and *S. bayanus* (*27*). However, in recent years, efforts have been made to select different yeast strains and also to use non-*Saccharomyces* yeasts, whether in mixed or pure cultures (*28*, *29*) aiming at a greater control of the fermentation process and of the characteristics of the product (*1*, *30*).

Some studies have also investigated the effects of additives, nutritional supplements, and vegetables, especially fruits, herbs and spices, in the preparation of mead in order to examine the fermentation kinetics and chemical characteristics of the product (*1*, *11*, *27*, *31*).

## IMPACT OF DIFFERENT INGREDIENTS AND ADDITIVES ON THE STAGES OF MEAD PREPARATION

**Fig. S1:**
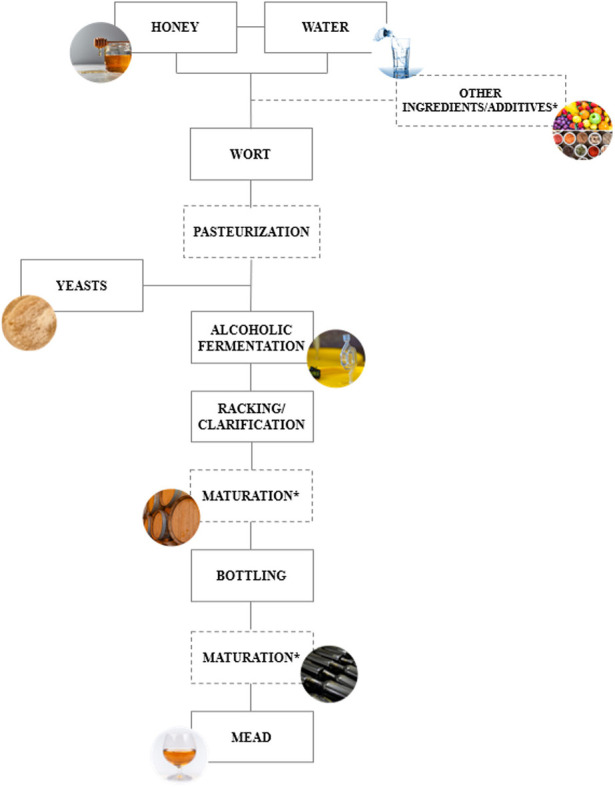
Flowchart showing the main steps in the preparation of mead. *Dotted rectangles represent optional steps in mead production. Adapted from (*9*) and (*32*)

In general, the mead preparation comprises some fundamental stages, which include prefermentation step (preparation of the wort), alcoholic fermentation step and post-fermentation step (clarification/bottling) (Fig. S1 (*9*, *32*)) (*7*). It is noteworthy that there may be changes in the initial and final stages of mead preparation depending on the available infrastructure and the characteristics that are desired for the product. Moreover, different ingredients and additives can be incorporated into the wort prior to the fermentation, while after racking maturation occurs optionally and can be carried out either before or after bottling.

### Prefermentation step

Honey can be diluted in water at different ratios according to the type of beverage that is intended to be obtained (*33*). For instance, the meads known as Półtoraki, Dwójniaki, Trójniaki and Czwórniaki are made using the following ratios: *V*(honey):*V*(water)=1:0.5, 1:1, 1:2 and 1:3 (*16*, *34*). It is important to point out that it is more usual to prepare worts with a total soluble solid (TSS) content around 20 to 25 °Brix (*32*, *35*) in order to obtain beverages with alcoholic strength from 10 to 13 %.

Although mead is obtained from a raw material that contains a high concentration of sugar, this alcoholic fermentation presents difficulties due to the chemical composition of honey, which lacks some nutrients necessary for yeast development, such as nitrogen and phosphorus (*6*, *9*). Honey contains a significantly low amount of nitrogen, which is an important element for adequate yeast growth (*36*). According to Morales *et al*. (*37*), substrates with nitrogen and phosphorus deficiency can prolong fermentation time, and thus yeast autolysis can occur, leaving the mead vulnerable to bacterial contamination. Therefore, after the honey has been diluted in water, additives and nutritional supplements that contain nitrogen, minerals, vitamins and acids can be added in the wort in order to stimulate yeast growth and, consequently, obtain better fermentation (*12*, *13*).

In view of these possible problems, it is important that the mead preparation is carried out under controlled conditions and with sufficient nutrients to obtain a quality product. In this sense, several studies have investigated the effect of the addition of prefermentative additives and supplements on the characteristics of mead (Table 1 (*1*, *6*, *9*-*11*, *13*, *27*, *28*, *32*, *38*-*44*)).

**Table 1:** Pre-fermentative additives used in making mead wort

Origin	Additive/supplement	Reference
Brazil	(NH_4_)_2_SO_4_, Mg and CaCO_3_	(*1*)
Brazil	(NH_4_)_3_PO_4_	(*6*)
Poland	CaCO_3_, (NH_4_)_3_PO_4_ and vitamin B_1_	(*9*)
Brazil	Yeast extract, malt extract, peptone, MgCl_2_, (NH_4_)_2_SO_4_ and (NH_4_)_2_HPO_4_	(*10*)
Brazil	SO_2_	(*11*)
USA	Fermaid-O (autolyzed yeasts of *S. cerevisiae*)	(*13*)
Poland	K_2_HPO_4_ and CaCO_3_	(*27*)
USA	Yeast extract and peptone	(*28*)
Spanish	C_4_H_6_O_6_, K_2_S_2_O_5_ and pollen	(*32*)
Portugal	K_2_C_4_H_4_O_6_, C_4_H_6_O_5_ and (NH_4_)_2_HPO_4_	(*38*)
Portugal	K_2_C_4_H_4_O_6_, C_4_H_6_O_5_ and (NH_4_)_2_HPO_4_	(*39*)
Portugal	(NH_4_)_2_HPO_4_	(*40*)
Portugal	Commercial nutrient, SO_2_ and C_4_H_6_O_6_	(*41*)
Portugal	K_2_C_4_H_4_O_6_, C_4_H_6_O_5_ and (NH_4_)_2_HPO_4_	(*42*)
Brazil	Commercial nutrient and SO_2_	(*43*)
Poland	K_2_HPO_4_ and CaCO_3_	(*44*)

Some compounds, such as (NH_4_)_2_HPO_4_ and K_2_C_4_H_4_O_6_, are added to the wort in order to increase the fermentative activity of yeasts, thus reducing fermentation time, besides acting on the production of volatile compounds, which are important for the aromatic complexity of the beverage (*10*, *38*-*40*).

Willey *et al*. (*13*) added autolyzed yeast-based supplements to mead and obtained fermented products with higher yeast assimilable nitrogen (YAN) contents, besides lower residual sugar contents and higher pH than of the sample without supplementation.

Preservatives are also often added to the wort, such as K_2_S_2_O_5_, which is used to prevent contamination by bacteria and other yeasts that can either interrupt fermentation or promote oxidative process (*32*). The same occurs when SO_2_ is added (*41*). It is noteworthy that before starting the fermentation, the wort can also undergo pasteurization in order to reduce its microbial load (*12*, *38*, *42*). However, Klikarová *et al*. (*45*) report that despite this step being helpful in a more controlled fermentation process, there is the possibility of it causing degradation of some thermolabile bioactive components of the wort (phenolic compounds, enzymes and vitamins), browning, oxidation and increased 5-hydroxymethylfurfural content. In this way, aiming to preserve the chemical compounds of the wort and its sensory characteristics, preservatives are often added before the fermentation of the mead.

Some organic acids are added to the mead wort in order to promote pH adjustment, such as tartaric (*41*) and malic acid (*38*, *39*, *42*), and thus provide a better balance between sweetness and acidity besides increasing the buffering capacity of the wort, thereby improving yeast activity throughout fermentation (*16*). In this sense, some plant species, as tamarind, pineapple, feijoa, uvaia and blackberry, have also been incorporated into the wort before mead fermentation (Table 2 (*1*, *3*, *6*, *9*-*11*, *27*, *31*, *43*, *44*, *46*-*48*)) to improve the characteristics of the product.

**Table 2:** Different ingredients used in the prefermentation steps of mead production

Origin	Type of honey	Yeast	Fruit/spice	Reference
Brazil	Floral	*S. cerevisiae* (ScST58)/*S. bayanus* (SbPB and SbPC)	Cowpea bean (0.5 and 30 g/L)	(*1*)
Italy	Honeydew Honey	*S. cerevisiae* (isolated from sweet wine and indigenous yeasts)	*Cannabis sativa* L. (0.25 and 0.50 %)	(*3*)
Brazil	Multifloral honey	*S. cerevisiae*	Moscato grape juice (10, 20 and 30 %)	(*6*)
Poland	Wild floral	*S. bayanus* (Safspirit fruit)	Dandelion syrup (10 %), chokeberry fruits (10 %) and grape seed powder (10 g/L)	(*9*)
Brazil	Floral	*S. cerevisiae* (AWRI 796)	Concentrated acerola pulp (10, 15, 20, 25 and 30 %)	(*10*)
Brazil	Wild floral	*S. bayanus* (SbPB)	*Ilex paraguariensis* (1 %)	(*11*)
Poland	Rapeseed honey	*S. bayanus* (Safspirit fruit)/*S. cerevisiae* (Safspirit malt)	Cornelia cherry (10 %)	(*27*)
Nigeria	No data	*S. cerevisiae*	*V*(honey):*V*(coconut milk)=1:1, 1:2, 2:1, 3:1 and 1:3	(*31*)
Brazil	Wild honey	*S. bayanus*	*Rubus* spp. cv. Tupy (10 %), *Acca sellowiana* Berg (10 %), *Eugenia pyriformes* Cambess (10 %)	(*43*)
Poland	Rapeseed honey	*S. bayanus* (Safspirit fruit)	Cornelian cherry juice (10 %)	(*44*)
Czech Republic	No data	No data	Cherry, blackcurrant, raspberry, herbs, nuts	(*46*)
Brazil	No data	*S. cerevisiae*	Tamarind pulp (10, 20 and 30 %)	(*47*)
Brazil	No data	*S. cerevisiae* (Montrachet)	Pineapple pulp (0, 10, 20 and 30 %)	(*48*)

Švecová *et al*. (*46*) conducted an evaluation of the Czech meads and noted that the samples containing cherry showed higher levels of citric acid (3130 mg/L), which were attributed to the fruit source and to the amount of the used fruit. According to Uzhel *et al*. (*49*), citric acid has been frequently added to fermented beverages to improve their antioxidant action and colour retention. On the other hand, Pereira *et al*. (*7*) noted low malic acid contents (mean values) in traditional meads, which suggests the need for the addition of this acid to worts, whether in its isolated or natural form, in order to stimulate the fermentation process. However, Romano *et al*. (*3*) observed an increase in the concentration of succinic acid during the fermentation of meads containing different parts of *Cannabis sativa* L. Succinic acid is a metabolite of alcoholic fermentation, which results in a rapid pH decrease in the first hours of the process, and strongly depends on the yeast strain and on the presence of nitrogenous compounds in the medium (*50*).

Meanwhile, Romano *et al*. (*3*) noted the production of acetic acid over five weeks of fermentation in samples containing hemp stalks (104.57 mg/L). Moreover, Kawa-Rygielska *et al*. (*9*) noted a significant increase in this acid over 16 days of fermentation in the meads with added aronia syrup (1070 mg/L), dandelion (1400.6 mg/L) and powdered grape seeds (1150 mg/L). However, the values these authors obtained were lower than those reported for the control sample (1700 mg/L). According to Sroka and Tuszyński (*50*), high osmotic pressure and unfavourable fermentation conditions can increase acetic acid synthesis by yeasts, leading to the accumulation of a higher concentration of this acid in the mead, which in turn can negatively affect the quality of the product.

Anunciação *et al*. (*47*) added tamarind pulp (10 %) to the mead wort, which resulted in an increase in the yeast cell viability during fermentation and also in an increase in the ethanol production. However, Amorim *et al*. (*10*) evaluated the effect of the addition of 0, 10, 15, 25 and 30 % of acerola pulp on the production of mead by *S. cerevisiae* AWRI796 and reported that the addition of increasing amounts of acerola pulp promoted a progressive increase in the cell growth of the fermentative yeast. Balogu and Towobola (*31*) noted that the addition of coconut milk to mead improved some fermentative parameters, especially of the sample prepared with honey wort (1200 mL) and coconut milk (600 mL), which showed higher attenuation (98.63 %), lower residual sugar content (3.01 g/L), and higher fermentation velocity (0.99) after 60 days of fermentation. According to Mascarenhas *et al*. (*48*), the addition of pineapple pulp to mead made it possible to obtain a beverage with 30 % more ethanol than the control sample. Araújo *et al*. (*1*) noted that a higher concentration of cowpea extract (30 g/L) had a stimulatory effect on the metabolic activities of yeasts, especially in relation to *Saccharomyces bayanus* (SbPB), thus resulting in a higher substrate consumption (90 %) and a greater ethanol production (15.5 %).

In a study conducted by Cavanholi *et al*. (*11*) on the mead with added yerba mate powder extract, obtained by cold infusion and by hot infusion, higher acidity values were noted in the samples containing yerba mate powder extract (60.0 to 60.5 mmol/L), and a higher content of total soluble solids (24.73 °Brix) in the wort with yerba mate powder extract obtained by hot infusion, which resulted in a higher alcohol content in the beverage (11.05 %).

### Alcoholic fermentation step

In the alcoholic fermentation step, the wort may be boiled to ensure aseptic conditions for fermentation (*34*). Furthermore, Starowicz and Granvogl (*51*) reported that controlled boiling of the wort can result in the mead with high aroma quality. However, temperature and boiling time are not well defined. Moreover, it is possible to note a discrepancy between the temperatures and boiling times in different studies (*34*, *52*).

The alcoholic fermentation step of mead is the biochemical process that occurs by the action of yeasts through the conversion of sugars from honey or other wort ingredients into ethanol and carbon dioxide (*36*). The yeasts that are mostly used in meads are those of the genus *Saccharomyces* (*14*, *53*), which must show high fermentative activity, high tolerance to osmotic pressure, and high concentrations of ethanol (*42*). However, recently the use of non-*Saccharomyces* yeasts, such as those of the *Torulaspora* genus, has been proposed for the preparation of either mixed or pure cultures aiming mainly to increase the aromatic complexity of the mead. Barry *et al*. (*28*) noted that the mixed culture of different *Torulaspora* strains (YH178 and YH179) together with *Saccharomyces cerevisiae* (WLP715) showed a good fermentative performance in less than 10 days. Moreover, mead fermented by *Torulaspora* strains showed better sensory characteristics, especially regarding its flavour.

It is important to highlight that the fermentation time for mead is quite variable and depends on the ingredients used in the wort, as well as on the dilution ratio and type of yeast (*50*). The temperature dictates the speed of the process (*36*), which is normally carried out between 22 and 25 °C and must be monitored periodically to minimize the risk of premature interruption of the fermentation (*7*, *32*, *33*). Some studies have been carried out aiming to optimize the fermentation time and increase the quality of mead. Roldán *et al*. (*32*) added pollen to the wort as a fermentation activator and noted an increase in the yield and fermentation efficiency of approx. 7 and 10 %, respectively, besides an increase in the volatile content and an improvement in the beverage sensory profile. Similar results were obtained by Kempka and Mantovani (*54*), who also added pollen to the mead (1 %) and noted a decrease in fermentation time, from 168 to 72 h, compared with the control sample (without pollen).

The end of fermentation is reached when the density of the mead remains constant, indicating the need for filtration to remove suspended particles that are deposited at the bottom of the fermentor (dregs). However, fermentation may also be interrupted in order to obtain mead with a sweet characteristic and lower alcohol content.

### Post-fermentation step

After fermentation, the racking of the mead (transfer of the wort from one container to another) is carried out (*12*) in order to remove the dregs that have settled on the bottom of the fermentor. The clarification of the mead can be carried out by centrifugation of the wort or by the addition of clarifying agents, such as bentonite, egg white, gelatin, casein, and others (*7*), in which case the insoluble solids of the beverage are removed by sedimentation. Silva *et al*. (*55*) investigated the effect of different clarifying agents (bentonite, banana peel flour and passion fruit peel flour) on the presence of biogenic amines in mead. These authors concluded that bentonite is a good binder for mead, since the mean values for the number of biogenic amines remained low and constant ​​during storage of the beverage. Moreover, the other clarifying agents used (banana peel flour and passion fruit peel meal) proved to be a viable alternative for the producer, as they showed a profile similar to that of the control (bentonite).

After clarification, the mead is bottled. The bottles must be stored in a cool environment, protected from light and without variation in temperature in order to maintain the chemical and sensory characteristics of the product (*56*). After that, the mead can be subjected to maturation, where the aromatic compounds in the beverage are developed. The maturation stage can take from several months to years. In some cases, in order to make the beverage more complex, the maturation of mead may take place in wooden barrels before bottling (*57*). The maturation of beverages in oak barrels promotes the integration of aromatic compounds, softening the structure of the beverage and balancing its flavour (*58*). However, other types of maturation techniques and wood can also be used in the maturation of mead. Fey *et al*. (*56*) conducted maturation of meads for 100 days using woodchips of European oak, jatobá (stinkingtoe) and jequitibá and noted a decrease in luminosity and an increase in the intensity of the yellow and red colours in the samples with the woodchips in comparison with the control (without woodchips) besides an increase in the content of some esters, especially ethyl acetate.

## BIOACTIVE POTENTIAL OF MEAD: EFFECT OF THE ADDITION OF DIFFERENT INGREDIENTS AND ADDITIVES ON THE PHENOLIC COMPOSITION OF THE BEVERAGES

Mead has been reported as a potentially bioactive beverage, mainly because of its profile of phenolic compounds and its antioxidant activity. The phenolic content of mead is strongly related to the ingredients that are used to produce it (*14*). It is worth noting that honey is an important source of bioactive compounds for mead since some phenolic compounds are transferred from the plants to the honey by the bees (*59*). However, the concentration of these compounds in mead depends on the origin and on the type of honey, besides the amount used in the preparation of the beverage (*60*). Although there are many studies on the phenolic profile and antioxidant activity of different types of honey, little information can be found regarding mead.

Phenolic compounds comprise a distinct class of secondary plant metabolites from different plant sources, such as fruits, cereals and herbs (*61*), which are rich in flavonoids, flavones, flavanones, isoflavones, anthocyanins, catechins, phenolic acids, phytoestrogens, tannins, stilbenes and curcuminoids (*62*). Phenolic compounds are potent antioxidants that reduce or even inhibit the propagation of oxidation reactions by scavenging the reactive form of oxygen (*60*).

As reported by Dhalaria *et al*. (*63*), the health benefits associated with the consumption of fruits and vegetables have drawn increasing interest from consumers. Thus, the inclusion of ingredients of vegetable origin in the mead can enhance the beverage’s bioactive properties, since Bednarek and Szwengiel (*52*) report that the antioxidant potential of mead can be improved by adding fruit juices and herbs. Table 3 (*9*, *27*, *46*, *52*, *60*) shows the phenolic composition of the mead containing different ingredients of vegetable origin.

**Table 3:** Phenolic profile of the mead containing different ingredients of plant origin

Mead	*γ*(phenolic compound)/(mg/L)	Reference
Chokeberry mead	Protocatechuic acid: 4.46 Flavonols: 1.78	(*9*)
Dandelion mead	Flavonols: 1.06	(*9*)
Grape seed mead	Gallic acid: 3.66 Procyanidins: 14.04	(*9*)
Yellow Cornelian cherry mead	Gallic acid: 2.30 *p*-Coumaric acid: 0.18 Loganic acid: 54.80 Ellagic acid: 0.205-O-caffeoylquinic acid: 1.20 Hydroxybenzoic acids: 4.10 Hydroxycinnamic acids: 1.60	(*27*)
Coral Cornelian cherry mead	Gallic acid: 1.20 *p*-Coumaric acid: 0.18 Loganic acid: 76.40 Ellagic acid: 0.20 5-O-caffeoylquinic acid: 1.80 Hydroxybenzoic acids: 3.40 Hydroxycinnamic acids: 2.30	(*27*)
Red Cornelian cherry mead	Gallic acid: 1.00 *p*-Coumaric acid: 0.18 Loganic acid: 48.80 Ellagic acid: 0.20 5-O-caffeoylquinic acid: 1.40 Hydroxybenzoic acids: 3.40 Hydroxycinnamic acids: 1.80	(*27*)
Sherry mead	Gallic acid: 0.805 Protocatechuic acid: 0.344 Gentisic acid: 0.039 Protocatechuic aldehyde: 0.029 4-Hydroxyphenylacetic acid: 0.057 Vanillic acid: 0.152 Caffeic acid: 0.087 Syringic acid: 0.038 Vanillin: 4.126 Ferulic acid: 0.224 Ethylvanillin: 0.075 *p*-coumaric acid: 2.335	(*46*)
Nut mead	Gallic acid: 0.100 Protocatechuic acid: 0.020 Protocatechuic aldehyde: 0.048 4-Hydroxyphenylacetic acid: 0.060 Caffeic acid: 0.081 Syringic acid: 0.088 Vanillin: 0.312 Ferulic acid: 0.059 Ethylvanillin: 0.063 *p*-coumaric acid: 0.097	(*46*)
Almond mead	Gallic acid: 0.084 Protocatechuic acid: 0.054 Gentisic acid: 0.022 Protocatechuicaldehyde: 0.027 4-Hydroxyphenylacetic acid: 0.054 Vanillic acid: 0.114 Vanillin: 40.403 Ferulic acid: 0.085 Ethylvanillin: 0.028 *p*-coumaric acid: 0.057	(*46*)
Blackcurrant mead	Gallic acid: 3.367 Protocatechuic acid: 1.525 Gentisic acid: 0.191 Protocatechuicaldehyde: 0.272 4-Hydroxyphenylacetic acid: 1.867 Vanillic acid: 0.724 Caffeic acid: 2.998 Syringic acid: 2.443 Vanillin: 1.297 Ferulic acid: 1.701 Ethylvanillin: 0.332 *p*-coumaric acid: 0.273	(*46*)
Raspberry mead	Gallic acid: 0.395 Protocatechuic acid: 1.089 Gentisic acid: 0.022 4-Hydroxyphenylacetic acid: 0.168 Vanillic acid: 0.742 Caffeic acid: 1.139 Syringic acid: 0.197 Vanillin: 0.280 Ferulic acid: 0.771 Ethylvanillin: 0.092 *p*-coumaric acid: 0.081	(*46*)
Herbal mead	Protocatechuic acid: 0.242 Gentisic acid: 0.015 Protocatechuic aldehyde: 0.027 4-Hydroxyphenylacetic acid: 0.248 Vanillic acid: 0.222 Caffeic acid: 0.797 Syringic acid: 0.066 Vanillin: 1.548 Ferulic acid: 1.919 Ethylvanillin: 0.091 *p*-coumaric acid: 0.110	(*46*)
Cherry mead	Gallic acid: 0.472 Protocatechuic acid: 0,600 Protocatechuic aldehyde: 0.238 4-Hydroxyphenylacetic acid: 0.494 Vanillic acid: 0.369 Caffeic acid: 1.317 Syringic acid: 0.271 Vanillin: 0.868 Ferulic acid: 2.418 Ethylvanillin: 1.496 *p*-coumaric acid: 0.109	(*46*)
Fruit juice, root extract and herbs	Vanillic acid: 0.12 Caffeic acid: 1.01 Syringic acid: 0.43 Ferulic acid: 0.21 *p*-coumaric acid: 3.92 Chlorogenic acid: 4.99 4-Hydroxybenzoic acid: 2.35 Apigenin: 0.04 Catechin: 0.01 Isoorientin: 0.05 Kaempferol: 0.10 Naringenin: 4.54 Orientin: 0.16 Quercetin: 0.32 Rutin: 1.75 Sinapic acid: 0.01 Tyrosol: 19.07	(*52*)
Multi-fruit juice	Gallic acid: 3.46 Protocatechuic acid: 0.74 Vanillic acid: 0.09 Caffeic acid: 0.33 Ferulic acid: 0.15 *p*-coumaric acid: 1.20 Chlorogenic acid: 0.24	(*60*)
Extracts from roots and herbs	Gallic acid: 7.56 Protocatechuic acid: 1.89 Vanillic acid: 0.24 Caffeic acid: 0.37 Ferulic acid: 0.30 *p*-coumaric acid: 0.41 Chlorogenic acid: 0.22	(*60*)
Herbs	Gallic acid: 0.73 Protocatechuic acid: 2.01 Vanillic acid: 0.29 Caffeic acid: 0.29 Ferulic acid: 0.20 *p*-coumaric acid: 0.93 Chlorogenic acid: 0.57	(*60*)
Blackcurrant juice and root spices	Gallic acid: 0.86 Protocatechuic acid: 1.38 Vanillic acid: 0.84 Caffeic acid: 0.87 Ferulic acid: 0.48 *p*-coumaric acid: 0.73 Chlorogenic acid: 0.22	(*60*)
Raspberry juice	Gallic acid: 1.36 Protocatechuic acid: 1.47 Vanillic acid: 0.15 Caffeic acid: 0.69 Ferulic acid: 0.50 *p*-coumaric acid: 0.19 Chlorogenic acid: 0.24	(*60*)
Rowanberry juice	Gallic acid: 1.15 Protocatechuic acid: 0.71 Vanillic acid: 0.33 Caffeic acid: 0.87 Ferulic acid: 0.49 *p*-coumaric acid: 0.04 Chlorogenic acid: 0.53	(*60*)
Chokeberry juice and root spices	Gallic acid: 0.78 Protocatechuic acid: 1.02 Vanillic acid: 0.33 Caffeic acid: 0.18 Ferulic acid: 0.11 *p*-coumaric acid: 0.17 Chlorogenic acid: 1.38	(*60*)
Root spices and herbs	Gallic acid: 0.80 Protocatechuic acid: 0.30 Vanillic acid: 0.12 Caffeic acid: 0.24 Ferulic acid: 0.21 *p*-coumaric acid: 0.03 Chlorogenic acid: 1.07	(*60*)
Sour cherry juice and root spices	Gallic acid: 0.55 Protocatechuic acid: 0.71 Vanillic acid: 0.40 Caffeic acid: 0.03 Ferulic acid: 0.15 *p*-coumaric acid: 0.20 Chlorogenic acid: 0.08	(*60*)
Plum must and root spices	Gallic acid: 1.14 Protocatechuic acid: 1.14 Vanillic acid: 0.40 Caffeic acid: 0.22 Ferulic acid: 0.10 *p*-coumaric acid: 0.18 Chlorogenic acid: 0.71	(*60*)

According to a study by Socha *et al*. (*60*), two groups of phenolic acids were identified in ten samples of commercial mead from Poland. These authors found that the plant species used in the preparation of the mead had a significant effect on the profile of phenolic acids, especially in the sample made with honey (in a 1:2 ratio of honey and water) and rowan juice (4.46 mg/L), and also in the sample of ’Kasztelanski’ mead (in a 1:1 ratio of honey and water) with added extract of roots and herbs (10.97 mg/L). Among the hydroxycinnamic acids, the highest measured amounts were of chlorogenic, caffeic, *p*-coumaric and ferulic acids, and among the hydroxybenzoic acids, the identified acids were gallic and protocatechuic (predominant among the analysed samples), besides vanillic acid. Zahrani *et al*. (*64*) reported that gallic acid is commonly found in several plant species, has different biological activities, and is considered a substance that contains antioxidant, antibacterial, antifungal, antiviral, anti-inflammatory and antidiabetic properties. However, in relation to protocatechuic acid, its antioxidant, anti-inflammatory and anti-apoptotic effects on animal tissues (mice) were proven in a study conducted by Habib *et al*. (*65*).

Švecová *et al*. (*46*) analysed 22 samples of commercial Czech meads and noted a great variability in the profile of phenolic compounds, which showed higher values for the samples containing additional ingredients (fruit juice, nuts, and herbal extracts) than the traditional meads, made only with honey and water. This result was especially evident in the mead with added black currant, which showed the highest content of phenolic compounds, with the emphasis on the concentrations of gallic (3.37 mg/L) and caffeic (2.99 mg/L) acids. It is noteworthy that caffeic acid can play an important role in the protection of different tissues and organs, protecting cell membranes from oxidative damage because of its ability to scavenge free radicals (*66*).

Adamenko *et al*. (*27*) investigated the effect of the addition of different varieties of Cornelian cherry on the polyphenol profile of meads and identified some compounds that belong to the groups of monoterpenes, phenolic acids and flavonoids. Among the monoterpenes, the iridoids, which until then had not been identified in the mead, were quantified. The iridoids from these monoterpenes have been reported to exert positive effects on the biological properties of the Cornelian cherry (*67*). The predominant phenolic acids were gallic (3.8 mg/mL) and chlorogenic (2.4 mg/mL) acids, showing higher concentrations in the mead fermented with the coral Cornelian cherry variety. Chlorogenic acid is a hydroxycinnamic acid that is widely present in plant species and has functional properties that are related to hypoglycaemic, hepatoprotective, antiviral, antibacterial and anti-inflammatory capacity (*68*). Besides these compounds, Adamenko *et al*. (*27*) also detected a different group of phenolic compounds that affect the biological properties and quality of foods, namely, the Q-3-glucuronide (Q-3-glcr), which was the only representative of the flavonols in the samples. On the other hand, anthocyanins were detected only in the mead with the addition of coral Cornelian cherry juice and red Cornelian cherry juice, with a predominance of those derived from pelargonidin. Anthocyanins have drawn increasing attention because of their preventive effect against some diseases, their ability to react with reactive oxygen species resulting from natural metabolic processes in plants or animals (*69*). Furthermore, according to Martín-Gómez *et al*. (*70*), the main health benefit properties of anthocyanins are their neuroprotective, cardioprotective, nephroprotective and ocular protection potential, as well as their anticarcinogenic and anti-inflammatory activity, among others.

In the study conducted by Kawa-Rygielska *et al*. (*9*), aronia syrup, dandelion and powdered grape seeds were separately added to the mead wort, and the phenolic compounds identified in the mead were hydroxycinnamic acids (derived from caffeic acid) and hydroxybenzoic acids (protocatechuic and gallic acid) as well as flavonols, procyanidins and flavanones. These authors noted that the highest concentration of phenolic acids was determined in the samples fermented with aronia syrup (52.9 mg/L of hydroxycinnamic acids and 4.39 mg/L of protocatechuic acid). Meanwhile, the mead with added powdered grape seeds stood out for its concentration of gallic acid (3.98 mg/L) and procyanidins (20.97 mg/L), which derived from the vegetable ingredient added to the wort. It is known that procyanidins have antibacterial, antioxidant and anti-obesity activities (*71*, *72*).

Bednarek and Szwengiel (*52*) investigated samples of commercial mead from Poland. The samples were saturated (heat treated) and unsaturated (non-heat treated), and also supplemented with fruit juice (raspberry, rowan and rosehip), root extract and herbs. These authors noted that the most abundant phenolic acid in the samples was chlorogenic acid, with average concentration of 4.99 mg/L. Naringenin was the flavonoid that had the highest concentration (4.54 mg/L), and is reported in other studies as the predominant flavonoid in Polish honeys (*73*). Naringenin is a flavanone usually present in citrus fruits. It has different pharmacological and biological properties regarding its antioxidant activity, which is related to anti-inflammatory and antitumour action, antimicrobial activity, and also fights the development of atherosclerosis (*74*, *75*). According to Smruthi *et al*. (*74*), naringenin is also present in glycosidic form, as naringin, which is responsible for the bitterness of the fruits.

Furthermore, the presence of tyrosol in mead was first reported in a study conducted by Bednarek and Szwengiel (*52*), who found that it was produced at concentration of 19.07 mg/L from tyrosine during fermentation. Tyrosol is a compound that is stable and less subject to autoxidation than other polyphenols, and *in vitro* and *in vivo* studies point to its biological potential related to antiatherogenic, cardioprotective and neuroprotective effects (*76*). This information may be interesting, since the polyphenols present in alcoholic beverages can have high bioaccessibility and are more easily absorbed by the intestine (*60*). However, so far there are no reports of studies that have evaluated the bioaccessibility of phenolic compounds in the mead.

It is important to emphasize that bioactive compounds from different sources added to mead tend to positively influence its sensory acceptability (*9*, *32*). However, it is important to evaluate the desirable concentrations of these compounds in the beverage.

## CHALLENGES AND PROSPECTS FOR THE PRODUCTION OF MEAD

Among the main challenges of the production of mead, the conditions of production stand out. This beverage is often produced either empirically or homemade, and producers many times come across problems related to the lack of standardization of the produced mead (*3*, *7*). Many of these problems are related to the harsh and adverse growth conditions to which yeasts need to respond and adapt. Another challenge for the productive sector is the standardization of mead in relation to its alcohol content, since mead is a beverage that shows a wide range of alcoholic content worldwide, generally between 8 and 18 % (*8*). Thus, depending on the ratio of honey and water used to make the wort, the incorporation of additional ingredients and the fermentation time, the obtained products will have completely different chemical and sensory characteristics. These differences reflect mainly on the sweetness parameters and on the perception of alcohol content, which are important characteristics for the consumer acceptance of beverages (*9*, *41*). Furthermore, Simão *et al*. (*77*) reported that although the technology patents related to mead are significant in number, there still is a demand for the diversification of this beverage.

Due to the slow scientific progress in this field, and taking into account the small amount of research studies on mead compared to other fermented alcoholic beverages (*e.g.* wine and beer), there is a possibility for research related to production issues, as well as the chemical quality, sensorial and bioactive aspects of the mead, which can and should be investigated.

## CONCLUSIONS

Despite being relatively simple, the mead preparation process demands caution in relation to different process parameters in order to obtain a high-quality beverage. The addition of different additives and ingredients greatly affects both the preparation process and the composition of the mead. Thus, there is a need for greater dissemination of technical and scientific knowledge for the production of this beverage on a large scale and under controlled conditions for its preparation. It is also noted that there is little data related to the bioactive potential of mead. This way, this review study serves as a motivation for further investigations aiming at the development of innovative products with potential benefits to human health. Therefore, research studies mainly related to the bioaccessibility of the bioactive compounds in mead have great scientific relevance, raising many questions that still need to be answered.
